# Dual Role of a Biosynthetic Enzyme, CysK, in Contact Dependent Growth Inhibition in Bacteria

**DOI:** 10.1371/journal.pone.0159844

**Published:** 2016-07-26

**Authors:** Soni Kaundal, Manju Uttam, Krishan Gopal Thakur

**Affiliations:** Structural Biology Laboratory, G. N. Ramachandran Protein Centre, Council of Scientific and Industrial Research-Institute of Microbial Technology, Chandigarh, India; University of Colorado Boulder, UNITED STATES

## Abstract

Contact dependent growth inhibition (CDI) is the phenomenon where CDI^+^ bacterial strain (inhibitor) inhibits the growth of CDI^−^strain (target) by direct cell to cell contact. CDI is mediated by *cdiBAI* gene cluster where CdiB facilitates the export of CdiA, an exotoxin, on the cell surface and CdiI acts as an immunity protein to protect CDI^+^ cells from autoinhibition. CdiA-CT, the C-terminal region of the toxin CdiA, from uropathogenic *Escherichia coli* strain 536 (UPEC536) is a latent tRNase that requires binding of a biosynthetic enzyme CysK (O-acetylserine sulfyhydrylase) for activation in the target cells. CdiA-CT can also interact simultaneously with CysK and immunity protein, CdiI, to form a ternary complex in UPEC536. But the role of CysK in the ternary complex is not clear. We studied the hydrodynamic, thermodynamic and kinetic parameters of binary and ternary complexes using AUC, ITC and SPR respectively, to investigate the role of CysK in UPEC536. We report that CdiA-CT binds CdiI and CysK with nanomolar range affinity. We further report that binding of CysK to CdiA-CT improves its affinity towards CdiI by ~40 fold resulting in the formation of a more stable complex with over ~130 fold decrease in dissociation rate. Thermal melting experiments also suggest the role of CysK in stabilizing CdiA-CT/CdiI complex as T_m_ of the binary complex shifts ~10°C upon binding CysK. Hence, CysK acts a modulator of CdiA-CT/CdiI interactions by stabilizing CdiA-CT/CdiI complex and may play a crucial role in preventing autoinhibition in UPEC536. This study reports a new moonlighting function of a biosynthetic enzyme, CysK, as a modulator of toxin/immunity interactions in UPEC536 inhibitor cells.

## Introduction

In most of the natural environments, microbes live in communities and adopt several survival strategies to compete or cooperate for limited resources [1]. In these microbial communities, bacteria have developed complex mechanisms to communicate with each other, either by secreting and sensing small chemical messenger molecules which involves phenomenon known as quorum sensing or by direct cell to cell contact. These bacterial communication systems help in coordinating multicellular behaviour like bioluminescence, biofilm formation, virulence, production of nanowire etc. and might also participate in discriminating ‘self’ or kin from ‘non-self’ or non-kin strains [2–6]. Direct cell to cell contact is probably a strategy by which bacteria can modulate the growth and behaviour of both siblings and competitors in close contact.

**Fig 1:**
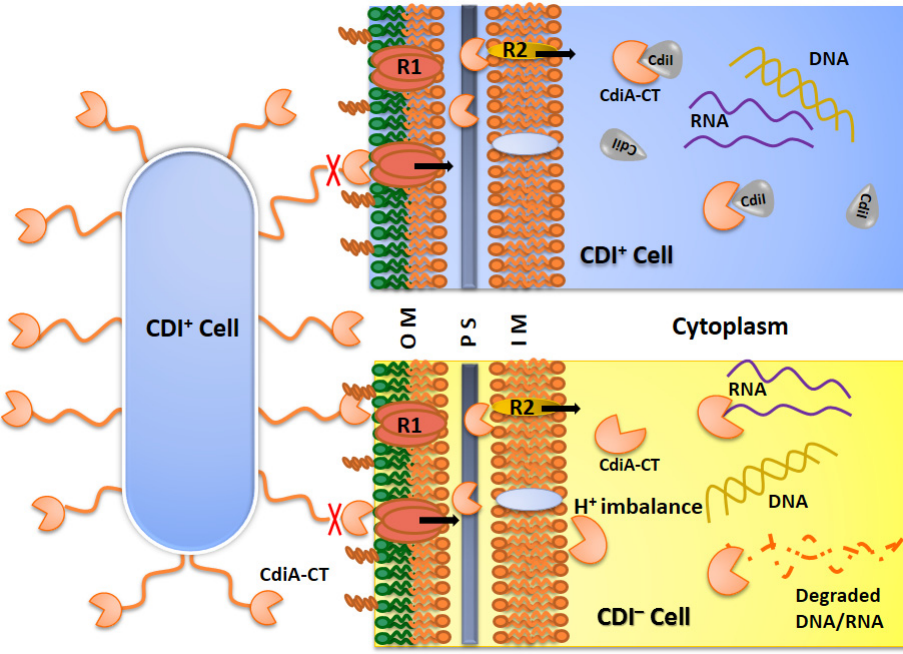
Schematic representation of mechanism of Contact Dependent Growth Inhibition in bacteria. CDI^+^ inhibitor cell expresses CdiA toxin on the cell surface. Upon coming in contact with receptor(s) present on the outer membrane of the cell, CdiA is cleaved and only toxic tip, CdiA-CT, enters the cell to reach molecular targets in the cytoplasm. CDI^+^ cells express CdiI which bind and neutralize cognate CdiA-CT to prevent autoinhibition. In CDI^−^cells CdiA-CT toxins act on different molecular targets like DNA, RNA, membrane etc. and causes growth arrest. This model is based on the published literature on CDI system from *E*. *coli* EC93 [7,9,20]. Abbreviations used: OM, Outer Membrane; PS, Periplasmic Space; IM, Inner Membrane; R1, Receptor 1; R2, Receptor 2

In 2005, Low and his co-workers coined a term “Contact Dependent growth Inhibition (CDI)” to describe a phenomenon where inhibitor cells require direct cell to cell contact to inhibit the growth of the target cells [7] (Fig 1). They further showed that CDI is mediated by *cdiBAI* gene cluster. CdiA and CdiB are components of two-partner secretion (TPS) system [8,9]. CdiB is a predicted β-barrel protein that facilitates export of CdiA across the outer membrane. CdiA is a large exotoxin, ranging from180–600 kDa, consisting of long filamentous hemagglutinin repeats and a toxic tip [10,11]. Multiple sequence analyses of the N-terminal region of CdiA from different species shows high sequence similarity, however, the C- terminal 200–300 residues, CdiA-CT, are highly divergent and predicted to have a wide range of catalytic and non-catalytic toxic activities [9,11]. CdiA is predicted to form a long filament from the surface of the inhibitor cell (CDI^+^) to bind the receptor on the target bacteria (CDI^–^) [9]. Since CdiA/CdiB shares sequence conservation with TPS systems so it is likely that CdiA protein folds outside the cell, as observed in TpsA protein [9,12]. But inhibitor cells face potential threat from re-entry of CdiA-CT toxin. These cells are prevented from autoinhibition due to the presence of an immunity protein, CdiI [7]. CdiI binds and neutralizes only the cognate CdiA-CT but not the heterologous ones [11]. These immunity proteins allow genetic counterparts of inhibitor cells to proliferate and outnumber their competitors [7]. Polymorphic nature of toxic tips also suggests that different bacteria may have different strategies to kill competitors or avoid the toxin neutralization from immunity proteins of the target cells [11,13]. Bioinformatics analyses suggest that CDI systems are widespread and are present in many plant, animal and human pathogens as well [5,11,13–15]. The CDI systems have so far been discovered in gram-negative bacteria and only acts upon closely related strains [7,16]. CDI systems have also been implicated in shaping biofilm structures to prevent non-self bacteria from entering the community [17,18]. In our study, we have investigated CDI system from a UPEC536, which causes recurrent urinary tract infection [19].

Recent studies suggest that CDI toxins exploit multiple target proteins to gain access to the target molecules localized in cytoplasm or membrane [9,21,22]. UPEC536 CDI system is more complex as compared to other CDI systems, as, besides the selection of target cell at the level of receptor-mediated uptake of a toxin, an additional step of selection was observed in the form of a biosynthetic enzyme, CysK (O-acetylserine sulfyhydrylase). CysK acts as a “permissive factor” that activates the otherwise inactive CdiA-CT and no significant growth inhibition was observed in the ΔCysK target cells [23]. CdiA-CT was shown to have tRNase activity in *in vivo* but purified toxin failed to show any activity in *in vitro* conditions. This led to the discovery of a host factor, CysK as an activator of tRNase activity of the CdiA-CT. Purified CysK activates CdiA-CT tRNase while CdiI (cognate immunity protein of CdiA-CT) is sufficient to block the resulting nuclease activity. Pull down assays have demonstrated that CdiA-CT forms a stable ternary complex with CysK and CdiI and both interacting partners bind non-overlapping sites on CdiA-CT. The C-terminal isoleucine residue of CdiA-CT is critical for CdiA-CT/CysK interactions as CdiA-CT^(ΔI3242)^ mutant is not able to bind CysK and lacks tRNase activity *in vitro* [23]. Given the fact that CdiA-CT, CdiI and CysK form a ternary complex within the inhibitor (CDI^+^) cells, the role of CysK and the basis of formation of this ternary complex in UPEC536 cells remain to be elucidated.

To address this important intriguing question, we investigated the role of CysK/CdiA-CT interactions in the context of inhibitor cell. We report for the first time the thermodynamic and kinetic parameters, oligomeric states and stoichiometry of proteins and protein-protein complexes involved in the CDI systems. We report a new moonlighting role of CysK in stabilizing CdiA-CT/CdiI immunity complex in UPEC536 and thus preventing autoinhibition. Taken together our findings and past reports, we propose that CysK plays two contrast roles–as a modulator of immunity complex in the inhibitor cells and as a toxin activator in the target cells, in UPEC536 mediated CDI. Our study establishes the significance of CysK in stabilizing inactive ternary complex in UPEC536 cells.

## Materials and Methods

### Cloning of Genes

In our experience, we have observed high toxicity of CdiA-CT toxin in *E*. *coli* as we could not get the functional gene cloned or expressed even in the presence of immunity protein gene. So, we used active site mutant CdiA-CT^536(H3193A)^, labeled CdiA-CT^(M)^, in our studies. This mutant reportedly lacks nuclease activity and retains gross structural architecture as evident from its tight association with the binding partner, CdiI [24]. Codon optimized synthetic genes *cdiA-CT*^*(M)*^ (Gly 3009-Ile 3242) and full-length *cdiI* were procured from GenScript USA Inc. *cdiA-CT*^*(M)*^, *cdiI* and *cysK* (amplified from *E*. *coli* K12 genomic DNA) gene products were cloned between NheI/HindIII, NdeI/XhoI and NcoI/XhoI restriction sites to yield pET28a-*cdiA-CT*^*(M)*^, pET28a-*cdiI* and pNIC28-Bsa4-*cysK* plasmids, respectively. The sequence of each construct was verified by DNA sequencing. All the recombinant proteins contained N-terminal 6x His-tag to aid purification.

### Purification of Proteins

Plasmid carrying desired gene(s) were transformed in *E*. *coli* BL21(DE3) cells and plated on LB agar plate having 50 μg/mL kanamycin and were incubated overnight at 37°C. Primary culture was prepared by inoculating single colony in 5 ml LB media and incubated overnight with constant shaking at 37°C. Primary culture was inoculated in 500 mL of LB media in shake flask and cells were induced by adding 0.3 mM IPTG at 0.6 OD_600_. Cells were further incubated at 18°C for overnight with constant shaking at 200 rpm. Cells were harvested by centrifugation at 7000g for 10 min and pellet was re-suspended in lysis buffer (20 mM HEPES pH 7.5, 250 mM NaCl, and a cocktail of protease inhibitors) followed by sonication. The supernatant was collected after centrifugation at 18000g for 45 min. The protein was purified using Co-NTA (Gold Biotechnology, USA) followed by gel filtration using Superdex 200 Increase 10/300 GL column (GE Healthcare) as per the standard protocols recommended by the manufacturers. The desired elution fractions were pooled and concentrated using ultrafiltration centrifugal devices (Pall Corporation). The purity and quality of the protein samples were analysed using SDS-PAGE and mass spectrometry. All the target proteins CdiA-CT^(M)^, CdiI and CysK could be purified using above-mentioned protocol to the purity of >95% as judged by the SDS-PAGE.

### Analytical Size Exclusion Chromatography (SEC)

Oligomeric state of purified proteins was analysed using the high resolution Superdex 200 Increase 10/300 GL column. 0.5 ml of concentrated samples (10, 20 and 30 μM) were loaded on the column which was pre-equilibrated with the running buffer (20 mM HEPES pH 7.5 and 250 mM NaCl). A constant flow rate of 0.5 ml/min was used and UV absorbance was monitored simultaneously at 280 nm, 254 nm and 220 nm at ~ 15°C. Standard calibration curve for the column was prepared using low molecular weight gel filtration calibration kit (GE Healthcare). To study protein-protein complexes, purified proteins were mixed (in varying molar ratios) and incubated at 4°C for 1 h before conducting SEC and analytical ultracentrifugation (AUC) experiments. The elution fractions were analysed using SDS-PAGE.

### Analytical Ultracentrifugation

AUC experiments were performed using Beckman-Coulter XL-A analytical ultracentrifuge equipped with a TiAn50 eight hole rotor to study the oligomeric state and stoichiometry of proteins and protein-protein complexes. Two-channel epon centrepieces (12 mm) and quartz windows were used for sedimentation velocity experiments. Three different concentrations of protein samples (10, 20 and 30 μM in the same buffer as used in the SEC experiments) were run and absorbance scans were recorded at 280 nm at every 3 or 4 min interval at 40,000 rpm at 15°C. Continuous distribution c(s) model was used to fit multiple scans at regular intervals with SEDFIT [25]. The solvent density (ρ) and viscosity (η) were calculated from the chemical composition of different proteins by SEDNTERP [26]. The values presented are the result of three independent experiments and the uncertainties were calculated using standard deviation.

### Isothermal Titration Calorimetry (ITC)

To determine the K_D_, stoichiometry and thermodynamic parameters of binding, ITC experiments were performed at 30°C using MicroCal VP-ITC (GE Healthcare). All the purified proteins were dialyzed against the buffer (20 mM HEPES pH 7.5, 150 mM NaCl), using dialysis membrane of 3500 MWCO. Degassing was performed using MicroCal ThermoVac (GE Healthcare) prior to the experiments. CdiA-CT^(M)^ (10 μM; sample cell) was titrated with CdiI (300 μM; syringe), and CdiA-CT^(M)^ (20 μM; sample cell) was titrated with CysK (120 μM dimer; syringe) with the constant stirring speed of 307 rpm. The injection volume and reference power were kept 6 μl and 5 μcal/sec respectively. To study the binding of CdiA-CT^(M)^/Cysk complex with CdiI, the complex CdiA-CT^(M)^/Cysk (5 μM heterotetramer; sample cell) was titrated with CdiI (100 μM; syringe) and for the binding of CdiA-CT^(M)^/CdiI with CysK, the complex CdiA-CT^(M)^/CdiI (30 μM heterodimer; sample cell) was titrated with CysK (150 μM dimer: syringe). One-site binding model was used to analyse CdiA-CT^(M)^/CdiI interactions. Since CysK is a dimer so based on the published literature, two binding site model was used to analyse protein-protein interactions involving CysK [27,28]. The binding isotherms were fit to determine apparent molar reaction enthalpy (Δ*H*), apparent entropy (Δ*S*), dissociation constant (*K*_D_) and stoichiometry of binding (*N*). To calculate ligand heat of dilution, experiments were performed under same experimental conditions except protein in the sample cell was replaced with the buffer. In all the calculations ligand heat of dilution was subtracted from the data. The data were analysed using Origin 6.0 software suite.

### Surface Plasmon Resonance (SPR)

To measure the kinetics of CdiA-CT^(M)^ binding to CdiI and CysK, SPR experiments were performed using Biacore 3000 at 30°C. CdiA-CT^(M)^ was diluted to 1 μM concentration in 10 mM sodium acetate (pH 5.0) and 723 response units (RU) were immobilized on the surface of the CM5 sensor chip. All experiments were carried out at a continuous flow rate of 20 μl/min using filtered and degassed buffer (10 mM HEPES pH 7.5, 150 mM NaCl,). The association and dissociation were observed for 300 s and 500 s for each complexes. The dissociation sensograms have been trimmed for better presentation of data. The analyte, CdiI was injected in a concentration gradient from 5 nM to 40 nM. The surface was regenerated by injecting 5 μl of 20 mM NaOH at flow rate of 5 μl/min. All the experiments were repeated at least three times at each concentration. The sensograms were analysed using BIA evaluation software 3.0 using a 1:1 Langmuir binding model of interaction. Whereas the analyte, CysK was injected in a concentration gradient from 0.4 μM to 6.4 μM (dimer concentration) followed by regeneration protocol as mentioned above. To study the kinetics of CdiA-CT^(M)^/CysK complex with CdiI, in first step 0.6 μM CysK (dimer) was passed over CdiA-CT^(M)^ to allow the formation of CdiA-CT^(M)^/CysK complex and then CdiI was injected in a concentration gradient from 2.5 nM to 40 nM followed by regeneration protocol as mentioned above. These experiments were conducted on a separate CM5 sensor chip where 760 RU of CdiA-CT^(M)^ were immobilized. The obtained sensograms were analysed using BIAevaluation software 3.0 using a local fitting for separate k_a_/k_d_ binding model of interaction.

### Thermal Stability Assay

To determine the effect of CysK binding on the thermal stability of the CdiA-CT^(M)^/CdiI complex we performed thermal melting assay using JascoJ-815 CD instrument. Experiments were performed using protein samples in concentration range of 3 to 5 μM dissolved in buffer containing 10 mM sodium phosphate, pH 7.4. Protein unfolding was observed by following the change in ellipticity at 220 nm wavelength as a function of temperature. The temperature was scanned from 25–95°C at a ramp rate of 1°C/min. The data were analysed using Origin 6.0 software.

## Results

### CdiA-CT^(M)^ and CdiI are monomer in solution and forms 1:1 stoichiometric complex

**Fig 2:**
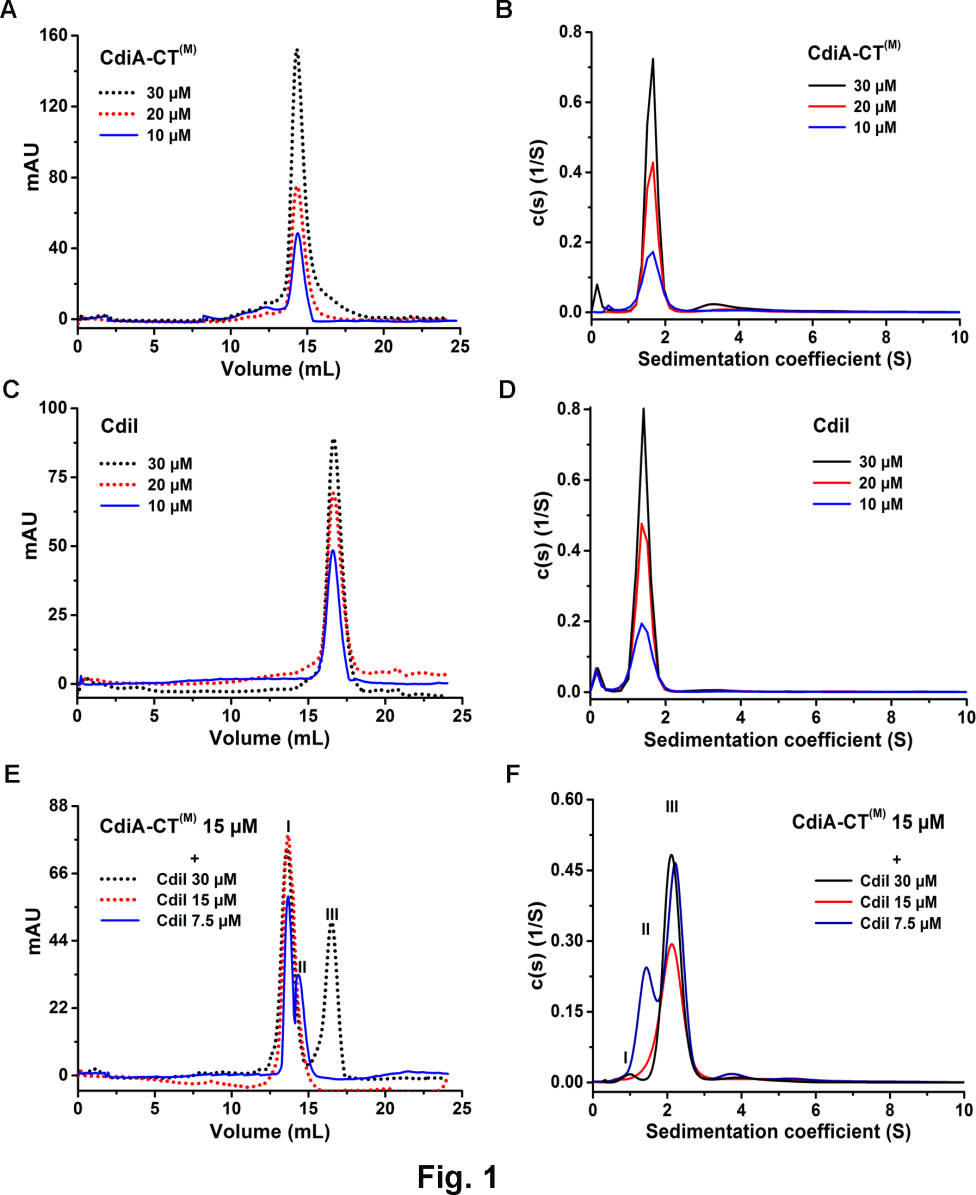
SEC and AUC analyses of the CdiA-CT^(M)^, CdiI and CdiA-CT^(M)^/CdiI complex. Sedimentation coefficient distribution c(s) are shown in the right panels and SEC profiles for the same samples are shown in the left panels. (A) and (B) Single predominant peak of CdiA-CT^(M)^ in SEC and AUC data respectively, suggest monodisperse samples. (C) and (D) CdiI also shows a predominant monomeric peak in both SEC and AUC respectively and no significant concentration dependent oligomerization was observed. (E) SEC analysis of CdiA-CT^(M)^/CdiI complex. Peaks I, II and III corresponds to CdiA-CT^(M)^/CdiI complex, CdiA-CT^(M)^ and CdiI respectively. Single major peak was observed when CdiA-CT^(M)^ and CdiI were mixed in 1:1 molar ratio. (F) AUC data suggests 1:1 stoichiometric complex of CdiA-CT^(M)^/CdiI (peak III). Under sub-stoichiometric conditions peak I correspond to excess CdiI and peak II corresponds to excess CdiA-CT^(M)^.

To determine the oligomeric state of CdiA-CT^(M)^ (26.9 kDa) and CdiI (16.8 kDa), a combination of SEC and AUC were used. SEC experiments were performed using Superdex 200 Increase 10/300 GL column as described in the materials and methods section. Purified CdiA-CT^(M)^ eluted as a major peak at 14.3 ± 0.04 mL which corresponds to a molecular weight of approximately ~51 kDa suggesting the apparent dimeric state of CdiA-CT^(M)^ in solution (Fig 2A). On the contrary, AUC data shows that major population of CdiA-CT^(M)^ exists as a monomer and a minor population (~5–10%) as a higher order oligomer i.e. tetramer (Fig 2B; Table 1). The population of this tetramer increases with increasing concentration of the protein. CdiA-CT^(M)^ is an elongated molecule as suggested by high frictional ratio observed in the AUC experiment. This elongated shape could explain the apparent dimeric state of CdiA-CT^(M)^ observed in the SEC experiments.

**Table 1:** Comparison of SEC and AUC data of proteins and protein-protein complexes indicating the oligomeric states and the binding stoichiometry. MW_obs_ and MW_cal_ are the observed and calculated molecular weights of the sample in kDa, respectively. The percentage population of the complexes mentioned in the parentheses corresponds to the samples where complexes were prepared by mixing equimolar ratio of the protein components.

Sample	MW_cal_ (kDa)	SEC		AUC			
		MW_obs_ (kDa)	Oligomeric State/ Stoichiometry	MW_obs_ (kDa) (% age)	Oligomeric State/ Stoichiometry	S_w_ (S)	Frictional ratio
CdiA-CT^(M)^	26.9	~51	Apparent Dimer	26.6 ± 0.07 (84%)	Monomer	1.7 ± 0.01	1.8 ± 0.01
CdiI	16.8	~20	Monomer	16.6 ± 0.02 (90%)	Monomer	1.5 ± 0.01	1.5 ± 0.02
CdiA-CT^(M)^/CdiI	43.7	~67	Heterodimer	41.2 ± 0.12 (70%)	Heterodimer	2.2 ± 0.13	1.89 ± 0.04
CysK	36.7	~70	Homodimer	73.7 ± 0.19 (84%)	Homodimer	4.3 ± 0.06	1.46 ± 0.01
CysK/CdiA-CT^(M)^	63.6	~230	Heterotetramer	126 ± 0.92 (80%)	Heterotetramer	4.1 ± 0.10	2.0 ± 0.02
CysK/CdiA-CT^(M)^/CdiI	80.4	~239	Heterohexamer	157.6 ± 0.52 (77%)	Heterohexamer	5.1 ± 0.14	1.90 ± 0.01

CdiI elutes at 16.6 ± 0.02 mL in SEC experiments which corresponds to ~20 kDa molecular weight, thus, suggesting monomeric state of CdiI in solution (Fig 2C). AUC data analysis also shows CdiI exists as a monomer in solution which is in agreement with SEC data (Fig 2D; Table 1).

To study the stoichiometry of CdiA-CT^(M)^/CdiI complex, the former was titrated with varying concentrations of the latter. When equal molar ratio of proteins were mixed, a single peak, corresponding to molecular weight of ~ 67 kDa, was observed in SEC, suggesting CdiA-CT^(M)^/CdiI forms a 2:1 complex (peak I, Fig 2E). Same complex, when analysed using AUC shows major population of molecular weight of 41.2 ± 0.12 kDa suggesting 1:1 stoichiometric heterodimer of CdiA-CT^(M)^/CdiI (peak III, Fig 2F; Table 1). To summarize both CdiA-CT^(M)^ and CdiI exist as a monomer in solution and forms a 1:1 stoichiometric complex. So, like CdiA-CT^(M)^ the elongated nature of the complex (frictional ratio 1.89, Table 1) migrates slowly during SEC experiment suggesting apparently higher molecular weight.

### CdiA-CT^(M)^ forms a 2:2 stoichiometric complex with CysK

**Fig 3:**
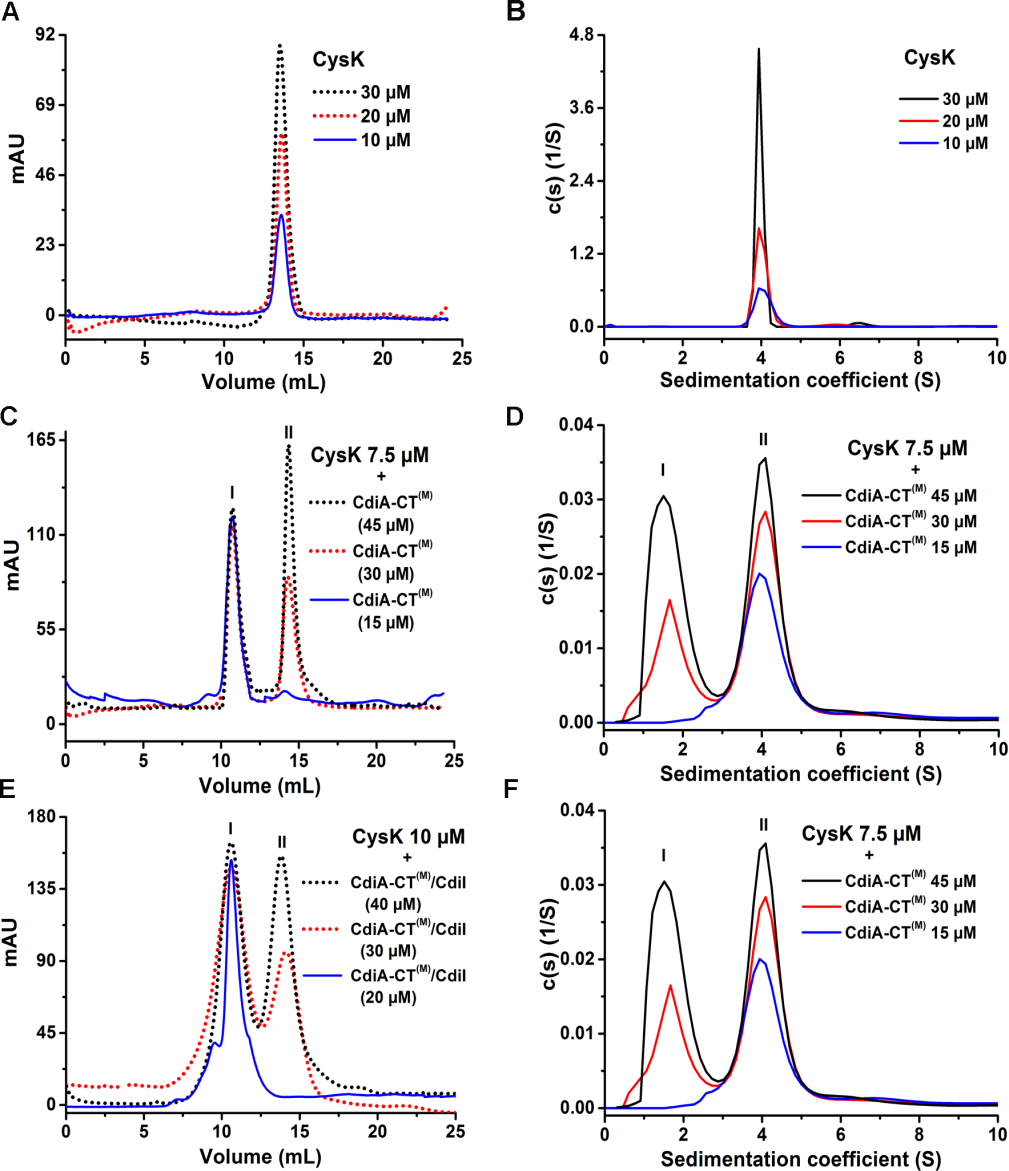
SEC and AUC analyses of the CysK, CdiA-CT^(M)^/CysK and CdiA-CT^(M)^/CdiI/CysK complexes. (A) and (B) Both SEC and AUC data suggests dimeric state of CysK. (C) CdiA-CT^(M)^/CysK complex elutes as a single peak when mixed 0.5:1 molar of CysK dimer:CdiA-CT^(M)^. Peak I, CdiA-CT^(M)^/CysK complex; peak II, excess CdiA-CT^(M)^ (D) AUC analysis suggests 2:2 stoichiometric complex of CdiA-CT ^(M)^/CysK. Peaks I and II corresponds to CdiA-CT^(M)^ and CdiA-CT^(M)^/CysK complex, respectively. (E) The stoichiometric homogenous ternary complex is formed when preformed CdiA-CT^(M)^/CdiI complex is mixed with dimeric CysK in 1:0.5 molar ratio. Peak I, CdiA-CT^(M)^/CdiI/CysK complex; peak II, excess CdiA-CT^(M)^/CdiI complex. (F) CdiA-CT^(M)^/CdiI/CysK forms 2:2:2 stoichiometric complex as suggested by AUC. Peak I, CdiA-CT^(M)^/CdiI complex; peak II CdiA-CT^(M)^/CdiI/CysK ternary complex. In all the above experiments dimeric CysK concentration is used.

We performed SEC and AUC experiments to study the oligomeric state of CysK and its binding stoichiometry with CdiA-CT^(M)^. Purified CysK (36.7 kDa) elutes as a single peak at around 13.6 ± 0.06 mL which corresponds to molecular weight of ~70 kDa thus suggesting that CysK exists as a dimer in solution (Fig 3A). AUC data also confirms dimeric state of CysK with the observed molecular weight of 73.7 ± 0.19 kDa (Fig 3B; Table 1). When 7.5 μM of CysK dimer was mixed with 15 μM of CdiA-CT^(M)^ a single peak was observed in SEC experiments suggesting CysK binds CdiA-CT^(M)^ with 2:2 stoichiometry. The complex elutes as a major peak at 10.7 ± 0.1 mL corresponding to molecular weight of ~230 kDa which is significantly higher than the expected molecular weight for this complex (Fig 3C). AUC data reveals that CdiA-CT^(M)^/CysK associate to form a complex with molecular weight of 126.0 ± 0.92kDa (~80% population) which matches with the expected molecular weight of 2:2 complex, suggesting CdiA-CT^(M)^/CysK forms a 2:2 stoichiometric complex (Fig 3D; Table 1).

### CysK/CdiA-CT^(M)^/CdiI forms a heterohexamer in 2:2:2 stoichiometry

CysK, CdiA-CT^(M)^ and CdiI form a stable ternary complex [23]. The oligomeric state and stoichiometry of this ternary complex are not known. So, we experimentally determined the oligomeric state of the ternary complex. Ternary complex elutes as a major peak at 10.6 ± 0.2 ml in SEC corresponding to molecular weight of ~239 kDa (Fig 3E). This single peak of ternary complex was observed when CysK dimer:CdiA-CT^(M)^/CdiI were mixed in 1:2:2 ratio. AUC data analysis revealed molecular weight of 157.6 ± 0.52 kDa complex which matches well with the expected molecular weight of 160 kDa corresponding to heterohexamer of the CysK/CdiA-CT^(M)^/CdiI complex in 2:2:2 stoichiometry (Fig 3F). High frictional ratio suggests the elongated shape of the ternary complex and possible reason for eluting early in SEC experiments.

### CdiA-CT^(M)^ binds CdiI with nanomolar affinity

**Fig 4:**
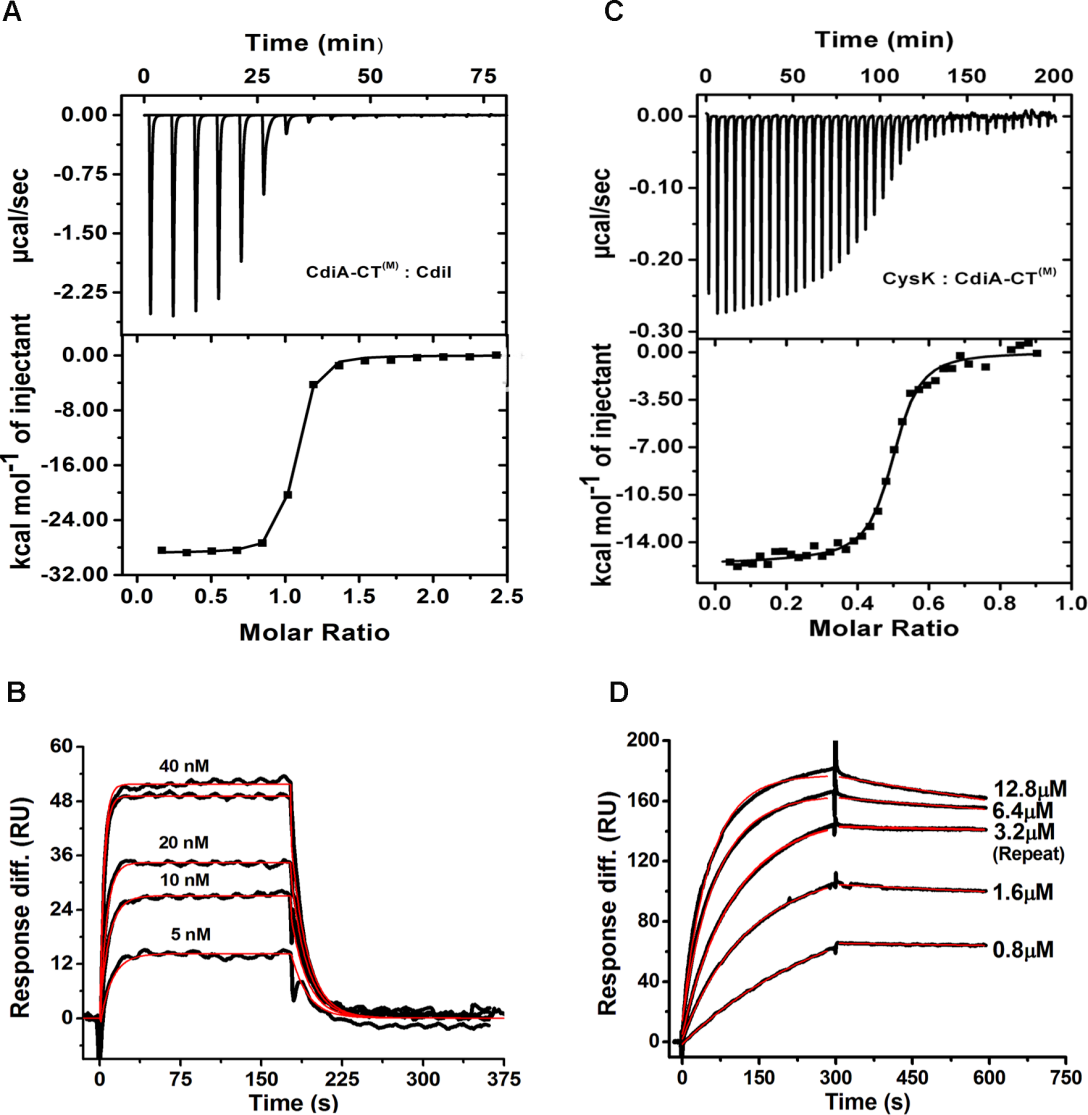
ITC and SPR data showing kinetics and thermodynamic parameters of protein-protein interactions. Representative isothermal calorimetric titration curve of **(A)** CdiA-CT^(M)^/CdiI **(C)** CdiA-CT^(M)^ and CysK. The top panel shows the calorimetric titration and bottom panel shows derived binding isotherm plotted versus the molar ratio of the titrant. The solid line in the lower panel is the best fit to the data using the nonlinear least squares regression algorithm. SPR analysis of binding of **(B)** CdiA-CT^(M)^/CdiI, **(D)** CdiA-CT^(M)^/CysK. Both raw sensograms (black lines) and fitted curves (red lines) are shown in the figure. These sensograms are representative of three sets of experiments that we carried out using four to five different concentrations of the analyte. In each figure, one representative sensogram for repeat run is also shown. (CdiA-CT^(M)^/CysK) complex. ITC data suggest that two monomers of CdiA-CT^(M)^ bind with one dimer of CysK.

CDI^+^ inhibitory cells are protected from autoinhibition by the presence of an immunity protein (CdiI) which physically interacts with the cognate toxin (CdiA-CT) [7]. To best of our knowledge, there is no study where thermodynamic and kinetic parameters of the toxin/immunity protein interactions involved in CDI systems have been reported. Though antitoxin perform diverse functions [29] they share one common function with immunity protein i.e. both neutralize/inactivate cognate toxin [7,11]. TA systems are produced within cells in response to different stress conditions and regulate gene expression CDI toxins are exotoxins and play an important role in competition. Since there are no reports of kinetic parameters of CDI systems so we compared our results with the bacterial toxin/antitoxin systems. So, in order to characterize the toxin/immunity protein interactions involved in CDI we carried out ITC and SPR experiments. The ITC data suggests 1:1 stoichiometry of binding with K_D_ value of 26.4 ± 2.1 nM (Fig 4A) whereas SPR data suggest K_D_ value of 12.8 ± 1nM (Fig 4B) for CdiA-CT^(M)^/CdiI binding at 30°C. K_D_ values estimated by the two different techniques are in close agreement. The stoichiometry of 1:1 as estimated from ITC data is in agreement with AUC data. This observed K_D_ value for CdiA-CT^(M)^/CdiI is significantly higher as compared to other reported toxin/antitoxin systems including RelB/RelE (K_D_: 0.33nM) [30], SpoII SA/ SpoII SB (K_D_: 0.46 nM) [31], BrnT/BrnA (K_D_: 0.67 nM) [32]. All these reported K_D_ values were measured using SPR. The thermodynamic data suggest a negative or favorable binding enthalpy (ΔH: –2.9 × 10^4^ ± 115.9 cal/mol) and unfavorable entropy factor (ΔS: –60.3 cal/mol/K) indicating a major role of hydrogen bonding and unfavorable conformational rearrangements in this binding event (Fig 4A; Table 2). SPR data analysis suggested high k_on_: 5.1 × 10^6^ M^–1^s^–1^ and unexpectedly high k_off_: 6.5 × 10^−2^ s^–1^ for this complex (Fig 4B). The k_off_ for CdiA-CT^(M)^/CdiI is significantly higher as compared to the other reported toxin/antitoxin systems including RelB/RelE (k_off_: 1.66 × 10^–4^s^–1^) [30], SpoII SA/SpoII SB (k_off_: 9.1 × 10^–4^s^–1^) [31], BrnT/BrnA (k_off_: 1.63 × 10^–4^s^–1^) [32]. Thus, it is evident that the lower K_D_ of CdiA-CT ^(M)^/CdiI complex is a consequence of significantly higher k_off_. On the basis of the kinetic data we speculate that as the toxin enters the CDI^+^ inhibitory cell, CdiI binds CdiA-CT quite rapidly but also apparently breaks apart swiftly as indicated by high k_on_ and k_off_.

**Table 2:** ITC and SPR data showing thermodynamic and kinetic parameters of protein-protein interactions involved in UPEC536 mediated CDI system. ITC data were fit using one-site binding model for CdiA-CT^(M)^/CdiI and two site binding model for rest of the complexes. The parameters for the second binding site are shown in the parentheses.

Protein samples	ITC				SPR		
	N	ΔH (cal/mol)	ΔS (cal/mol/K)	K_D_ (nM)	K_D_ (nM)	k_on_ (M^–1^s^–1^)	k_off_ (s^–1^)
CdiA-CT^(M)^ + CdiI	0.993	-2.9 × 10^4^ ± 115.9	-60.3	26.4 ± 2.1	12.8 ± 1	5.1 × 10^6^	6.5 × 10^−2^
CdiA-CT^(M)^ + CysK	0.494	-5618 ± 167.9 (-2.02 × 10^4^ ± 145.9)	14.0 (-34.5)	79.3 ± 8.9 (89.2 ± 10.1)	32 ± 4	6.2 × 10^3^	1.4 × 10^−4^
CysK/CdiA-CT^(M)^ + CdiI	1.892	-2.67 × 10^4^ ± 1.28 × 10^3^ (-2.42 × 10^4^ ± 1.18 × 10^3^)	-46.6 (-38.9)	1.3 ± 0.5 (1.1 ± 0.3)	0.3 ± 0.08	1.5 × 10^6^	5.0 × 10^−4^
CdiA-CT^(M)^/ CdiI + CysK	0.512	-1.1 × 10^4^ ± 157.9 (-2.67 × 10^4^ ± 267.9)	-0.9 (-31.1)	12.0 ± 1.9 (13.4 ± 2.3)			

### CdiA-CT^(M)^ and CysK binds with nanomolar affinity

CysK is required for the nuclease activity of CdiA-CT and both the proteins interact physically to form an activated complex [23]. We used ITC and SPR to determine affinity and kinetics of this toxin/activator (CdiA-CT^(M)^/CysK) complex. ITC data suggest that two monomers of CdiA-CT^(M)^ bind with one dimer of CysK with K_D1_ of 79.3 ± 8.9 nM and K_D2_ of 89.2 ± 10.1 nM suggesting similar affinities for both the binding sites (Fig 4C). Calculated changes in enthalpy (ΔH_1,2_) and entropy (ΔS_1,2_) are summarized in Table 2. SPR data also suggests similar high affinity complex with K_D_ of 32 ± 4 nM (Fig 4D). The stoichiometry of 2:2 as estimated from the ITC data is in agreement with the AUC data. SPR data analysis suggests slow rates of association (k_on_: 6.2 × 10^3^ M^–1^s^–1^) and dissociation (k_off_: 1.4 × 10^−4^ s^–1^) as compared to CdiA-CT^(M)^/CdiI binding. Low dissociation rate is advantageous for the toxin as upon activation enzyme may remain active for longer period consequently causing growth arrest in the target cell.

### CysK/CdiA-CT^(M)^ complex binds CdiI with ~40 fold higher affinity as compared to CdiA-CT^(M)^

**Fig 5:**
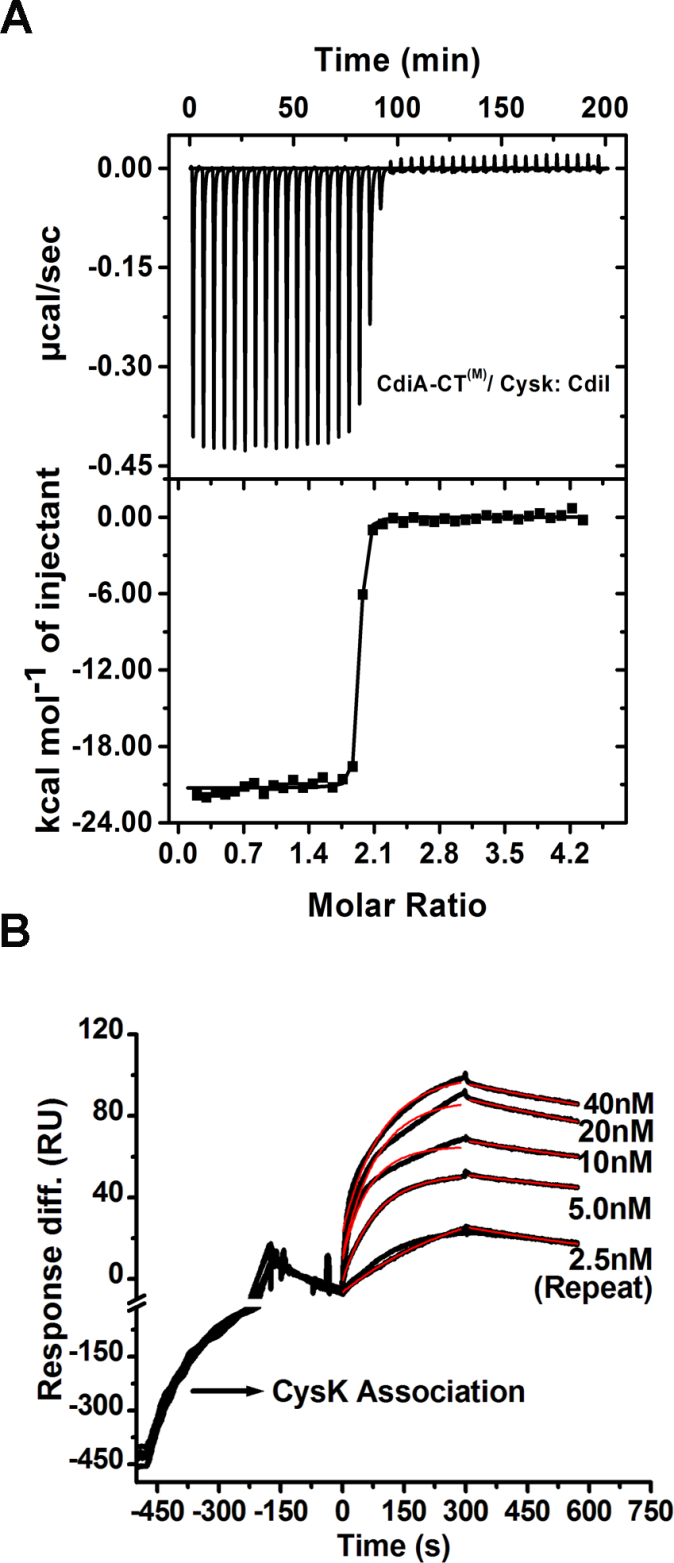
Interactions of CdiA-CT/CysK and CdiI studied using ITC and SPR. (A) ITC data suggests increased affinity of the CdiA-CT^(M)^/CysK complex for CdiI. (B) SPR data also suggests high affinity as a consequence of decreased dissociation rate. Both raw sensograms (black line) and fitted curves (red line) are shown in the figure. For better visualization of CysK/CdiA-CT^(M)^ complex and CdiI interactions scale is broken at –10 RU on the y-axis and at –200 s on the x-axis. The CdiI association starts at 0 s and ends at 300 s followed by dissociation phase of 500 s but shown only up to 300 s.

Both CdiI and CysK can simultaneously bind CdiA-CT and form a ternary complex which reportedly lacks nuclease activity [23]. To understand the possible role of CysK in this ternary complex in CDI^+^ inhibitory cells we investigated CdiA-CT/CdiI interactions by ITC and SPR. To our surprise, ITC data suggested that two CdiI monomer binds preformed CdiA-CT^(M)^/CysK heterotetramer complex with significantly lower K_D1_: 1.3 ± 0.5 and K_D2_: 1.1 ± 0.3 nM (Fig 5A) which is ~25 fold lower than CdiA-CT^(M)^/CdiI complex. SPR data also suggest ~40 fold lower K_D_ (0.33 ± 0.08 nM) of the ternary complex compared to CdiA-CT^(M)^/CdiI complex (Fig 5B). The thermodynamic parameters of this association are summarized in Table 2. The k_on_ and k_off_ of this binding are 1.5 × 10^6^ M^–1^s^–1^ and 5.0 × 10^−4^ s^–1^, respectively (Fig 5B). The k_off_ is ~130 fold lower as compared to that observed for the CdiA-CT^(M)^/CdiI complex. Nonetheless, there is a ~3 fold decrease in the association rate as well. These results indicate that ternary complex is much more stable than CdiA-CT^(M)^/CdiI complex which is majorly the consequence of the decrease in the dissociation rate.

### Binding of CdiI increases the affinity of CysK towards CdiA-CT^(M)^

**Fig 6:**
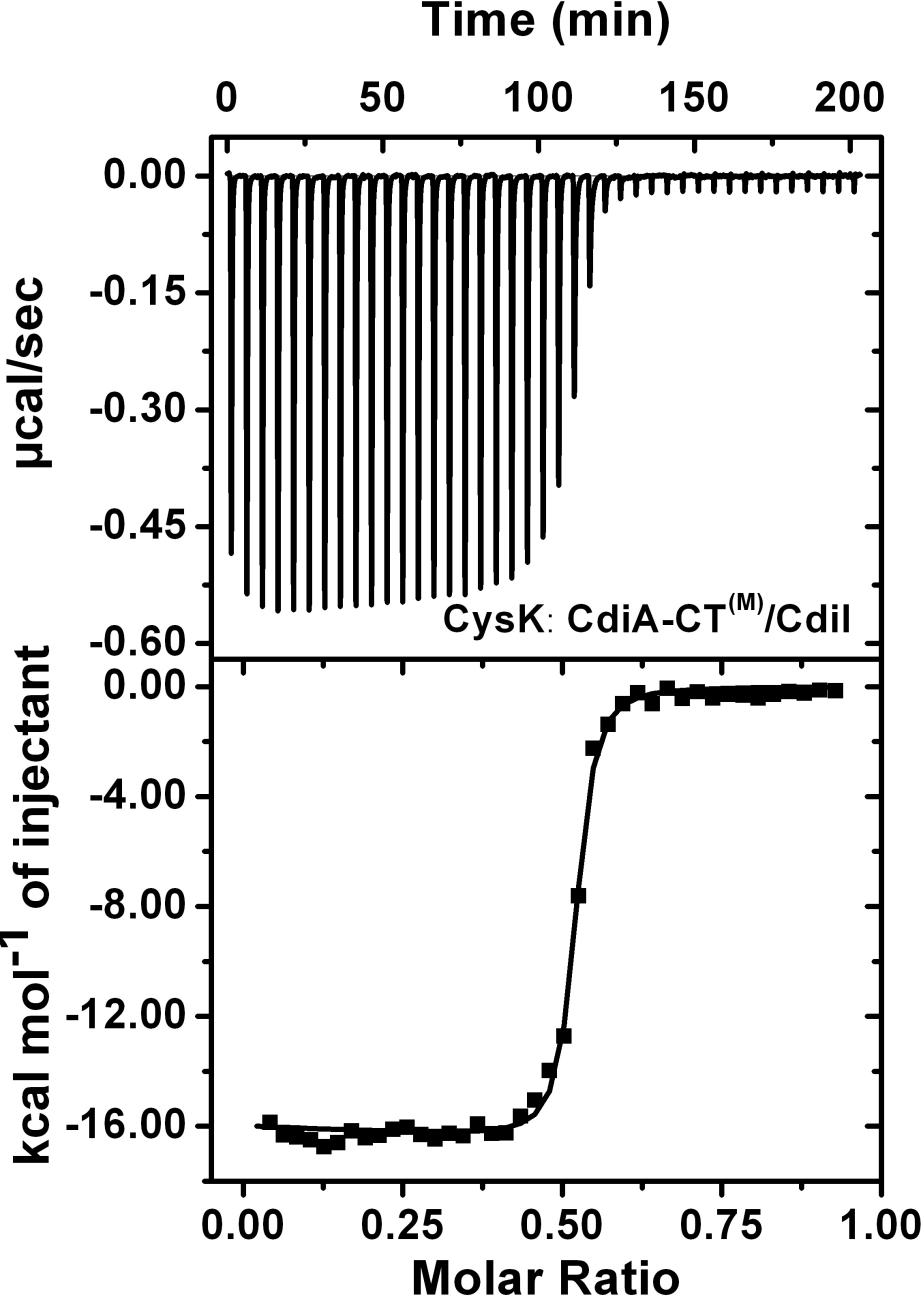
Interactions of CdiA-CT^(M)^/CdiI and CysK studied using ITC. ITC data suggests increased affinity of CysK for CdiA-CT^(M)^/CdiI complex.

It is not clear whether ternary complex formation follows a specific binding sequence. So, we studied the affinity of CdiA-CT^(M)^/CdiI complex with CysK. As CdiI has a high dissociation rate (Fig 4) so we could not use SPR for this study. We used ITC where the preformed complex of CdiA-CT^(M)^ and CdiI was titrated with CysK. ITC data suggests a high affinity with K_D1_ of 12.0 ± 1.9 and K_D2_ of 13.4 ± 2.3 nM (Fig 6) which is ~5 fold lower as compared to that observed for CdiA-CT^(M)^. This suggests that CysK can bind both CdiA-CT^(M)^ and CdiA-CT^(M)^/CdiI complex albeit it has a higher affinity for the latter.

### CysK significantly improves thermal stability of CdiA-CT^(M)^ and CdiA-CT^(M)^/CdiI

**Fig 7:**
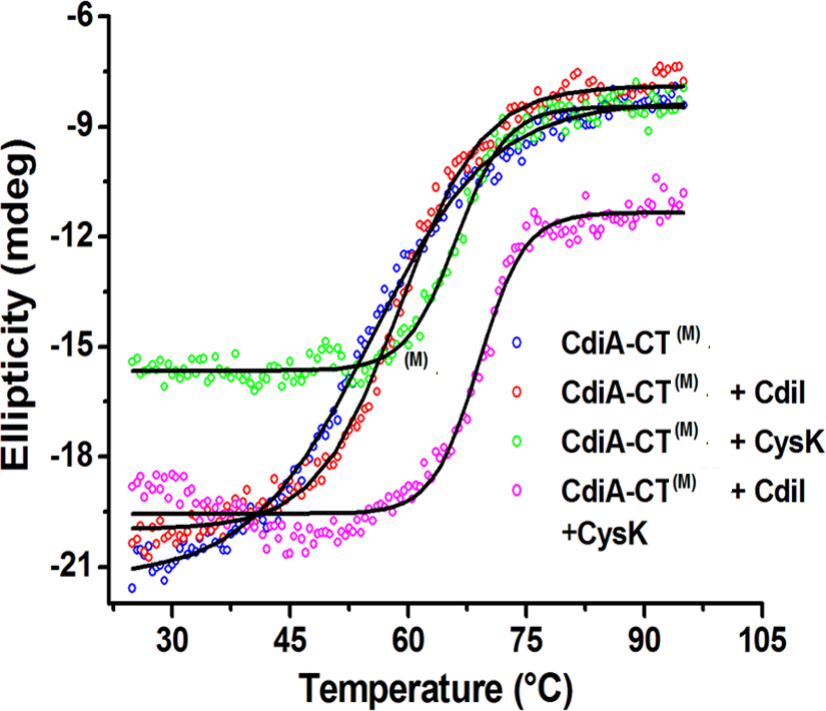
Binding of CysK stabilizes the CdiA-CT^(M)^/CdiI complex. Thermal stability of CdiA-CT^(M)^ and other complexes was determined using CD melting studies. All the protein samples show two state melting curve. CysK stabilizes CdiA-CT^(M)^ and CdiA-CT^(M)^/CdiI complex by ~10°C.

To compare the effect of CysK binding on the thermal stability of CdiA-CT^(M)^/CdiI binary complex, we monitored protein unfolding with increasing temperature using CD. Thermal melting experiments suggest that CdiA-CT^(M)^/CdiI complex exhibit two state melting curve with melting temperature (T_m_) of 58.9 ± 0.2°C. The ternary complex also exhibits two state melting curve showing cooperative unfolding and having T_m_ of 69.0 ± 0.2°C which is ~10°C higher than the CdiA-CT^(M)^/CdiI complex (Fig 7). On the other hand, CdiA-CT^(M)^ has a T_m_ of 55.2 ± 0.2°C and association of CysK to CdiA-CT^(M)^ increases thermal stability by 10°C as CdiA-CT^(M)^/CysK complex has T_m_ of 66.09 ± 0.15°C. Also, binding of CdiI stabilizes CdiA-CT^(M)^ by ~4°C. This data suggests that CdiI binding stabilizes CdiA-CT^(M)^ but CysK binding significantly enhances the thermal stability of both CdiA-CT^(M)^ and CdiA-CT ^(M)^/CdiI complex. The thermal stability results are in agreement with other CDI toxin/immunity complexes from *E*. *coli* EC869 and *Burkholderia pseudomallei* 1026b where binding of immunity protein resulted in improved thermal stability of the toxin/immunity complexes [33].

## Discussion

CDI is an interesting mode of direct cell to cell communication in bacteria that provides a survival advantage to CDI^+^ cells over CDI^−^ cells. Pioneering work by Low’s and Hayes’s groups has led to the discovery and detailed molecular dissection of the mechanism(s) involved in the CDI systems [7,9,11,21,24,34]. Body of the literature generated over a decade of research by these groups suggests that CDI involves several steps: i) expression of CdiA protein and its export to surface by CdiB in inhibitor cells, ii) cell to cell contact with the target cell, iii) receptor mediated CdiA-CT toxin uptake by target cell, iv) activation of the toxin in host by ‘cell permissive factor’ (optional step), v) target cell growth arrest, vi) protection of self by expression of immunity protein and hence promoting ‘self’ or ‘kin selection’. The presence of CdiA specific receptor protein on the target cell acts as a first line of the target selection [9,21]. Discovery of the role of CysK as a host ‘permissive factor’ has added one more distinguishing step to the existing complexity in the target selection in in UPEC536 mediated CDI system. Based on the presence of conserved C-terminal isoleucine in many predicted CDI toxins, it has been proposed that host factor(s) may act as a second line of target selection [23,35]. However, it is intriguing why inhibitor cells chose conserved biosynthetic enzyme CysK as cell permissive factor which is also present in its own genetic makeup as well? Interestingly, CysK and CdiI bind simultaneously to CdiA-CT to form a stable inactive ternary complex in CDI^+^ inhibitory cells [23]. Though ternary complex is inactive, the role of CysK in this ternary complex was not clear.

We used a combination of biophysical experiments to address this important question. In the CDI^+^ inhibitory cell, the binding sequence of CdiI, CysK and CdiA-CT to form ternary complex is not defined. The ternary complex can be formed by two distinct sequence of interactions: 1) CdiI binding to the preformed CysK/CdiA-CT complex and 2) CysK binding to the preformed CdiA-CT/CdiI complex. We studied both possible binding sequence events. To our surprise, CysK bound CdiA-CT shows ~40 fold increased affinity for CdiI as compared to free CdiA-CT. This increase in stability is mainly due to ~130 fold reduction in dissociation rate. We propose that this reduced dissociation rate is beneficial for inhibitor cells as the ternary immunity complex captures the toxin in an inactive state for the extended period and hence offering better protection. Our data suggest, that UPEC536 cells adopted allosteric/inactivation strengthening mechanism, by increasing the affinity of toxin/immunity protein interactions upon binding of CysK, to neutralize toxic activity of CdiA-CT/CysK activated complex. In this mode of inhibition, CdiI might be exploiting conformational changes upon CysK binding as a selective pressure to preferably bind activated CdiA-CT/CysK complex and form a high affinity inactive ternary complex with slower dissociation rate. Since CdiI dissociates rapidly from CdiA-CT/CdiI complex and dissociates at significantly much lower rate from the ternary complex hence, CdiI is engaged in preferably neutralizing active toxin/activator complex.

When we investigated the second binding sequence we observed that binding of CysK to preformed CdiA-CT /CdiI complex shows higher affinity as compared to CdiA-CT. These results suggest that CysK in CDI^+^ inhibitor cells may preferably associate with CdiA-CT/CdiI complex than with toxin alone hence providing an additional survival benefit. Another similar interesting case of regulation of cysteine metabolism mediated by CysK/CymR interactions has been reported in *Bacillus subtilis*. CymR, master regulator of cysteine metabolism in *B*. *subtilis*, forms a stable complex with CysK. Binding of CysK to CymR increases the stability of CymR/*ytll* promoter DNA by ~7 fold mainly by reducing the dissociation rate, as inferred from the SPR studies [36]. CysK is conserved in bacteria and plants and is reportedly involved in several moonlighting functions where the interacting partner usually have conserved C-terminal isoleucine residue [35]. Though we have studied the role of CysK in UPEC536 strain but there are several toxin/immunity protein systems where the toxins have terminal Ile residue [35] so we believe CysK may play similar roles in those CDI modules as well. In future, it will be interesting to study binding kinetics of such systems as well. Thermal melting studies revealed that CysK binding also increases the thermal stability of the CdiA-CT and CdiA-CT/CdiI complex by ~10°C. So, CysK binding provides kinetic as well as thermal stability to the CdiA-CT/CdiI immunity complex.

We also studied stoichiometry and kinetics of CdiA-CT /CysK interactions as this complex is common in both target and inhibitor cells. CdiA-CT binds CysK with K_D1_ of 79.3 ± 8.9 and K_D2_ of 89.2 ± 10.1 nM and 32 ± 4 nM as determined by ITC and SPR respectively. Interestingly, both k_on_ and k_off_ are significantly lower as compared to CdiA-CT/CdiI complex. Lower k_off_ is advantageous for the toxin as once activated, it may remain functional for a longer period, before the activated complex falls apart, causing prolonged damage to the target cell. AUC data suggests that CysK is predominantly dimer and CdiA-CT is predominantly monomer in solution. Based on the structural information available on close sequence homologs of CysK, the two CdiA-CT binding sites are positioned on the same face of the CysK dimer [37]. So, either two molecules of CdiA-CT can bind CysK dimer or binding of one CdiA-CT molecule may sterically occlude binding of the other molecule. To check stoichiometry of the complex we performed AUC experiments which suggest that CysK and CdiA-CT associate to form 2:2 stoichiometric heterotetramer in solution. Though the mechanism of the toxin activation is not yet clear, but based on the data presented here we speculate that CysK brings two molecules of CdiA-CT in close proximity and along with subtle conformational rearrangements may activate CdiA-CT.

**Fig 8:**
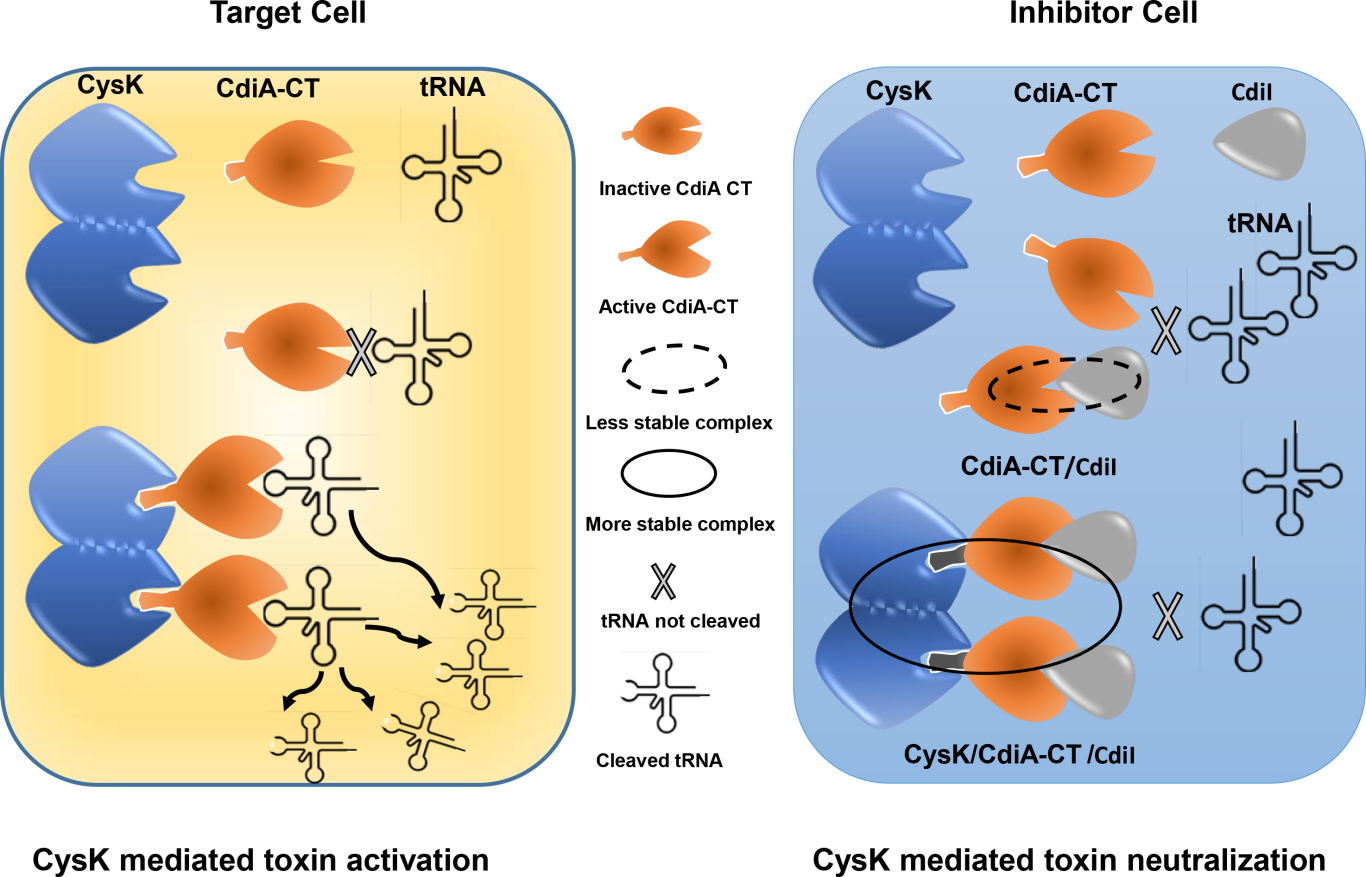
Proposed model for the dual role of CysK in UPEC536 mediated CDI. The left panel shows CysK mediated toxin activation in target cell where CysK dimer binds CdiA-CT and activates tRNAase activity causing growth arrest [23]. The right panel shows the moonlighting role of CysK as a modulator of CdiA-CT ^(M)^/CdiI in the CDI^+^ inhibitory cells, as proposed in the present study. Binding of CysK to CdiA-CT stabilizes CdiA-CT/CdiI immunity complex, primarily by decreasing dissociation rate, to prevent autoinhibition in UPEC536 inhibitor cells.

To summarize, this study demonstrates a distinct function of CysK in UPEC536 cells. We propose that CdiA-CT/CysK interactions, besides toxin activation, might have also evolved to enhance inherently weaker CdiA-CT/CdiI interactions to ensure better survival of inhibitor cells upon entry of CdiA-CT from the neighbouring CDI^+^ UPEC536 cells (Fig 8). This discovery adds one more role to the growing list of moonlighting functions mediated by CysK [35,36,38]. In future, more detailed structural/biophysical studies are needed to unravel the mechanism by which CysK or similar moonlighting proteins enhance the stability of protein-protein or protein-DNA complexes to perform some important biological functions.
